# Isolated rheumatic tricuspid valve regurgitation: it is only rare not just a myth: rare case report

**DOI:** 10.1186/s43044-024-00487-1

**Published:** 2024-05-07

**Authors:** Vemmy Lian Saputri, Valerinna Yogibuana

**Affiliations:** Department of Cardiology and Vascular Medicine, Faculty of Medicine, Dr. Saiful Anwar General Hospital, Universitas Brawijaya, Jl. Jaksa Agung Suprapto No. 2, Malang, East Java 65112 Indonesia

**Keywords:** Latent rheumatic heart disease (RHD), Isolated rheumatic tricuspid regurgitation (IRTR), Severe tricuspid regurgitation, Echocardiography, Diagnosis approach, Case report

## Abstract

**Background:**

Isolated rheumatic tricuspid regurgitation (IRTR) is a rare condition that can manifest as right heart failure (RHF) and pulmonary hypertension (PH) symptoms. Diagnosing and treating IRTR in cases of latent RHD can be a challenge and crucial for future research to establish new guidelines for echocardiography in RHD that focus not only on the mitral and aorta but also the tricuspid valve.

**Case presentation:**

A young female patient with clinical symptoms of RHF suspected IRTR due to latent RHD from echocardiography. Echocardiography revealed significant thickening and calcification of all tricuspid valve (TV) leaflets, with partial prolapse posterior leaflet and severe tricuspid regurgitation (TR) with a high probability of PH, no significant anatomical and functional abnormality pulmonary valve (PV), mitral valve (MV), and aortic valve (AV). She was administered daily doses of Ramipril, bisoprolol, spironolactone, and furosemide. Although she received therapy, she persisted in suffering dyspnea when doing mild physical activity (NYHA functional class III). She was admitted to the surgical conference, due to our center’s limitation of percutaneous intervention for valve replacement, and she was approved to undergo tricuspid valve replacement (TVR) surgery.

**Conclusions:**

Echocardiography plays a crucial role in identifying latent RHD. Isolated rheumatic TR shows echocardiographic results similar to rheumatic mitral regurgitation, except for the presence of a high-velocity jet. Diuretics temporarily slow symptoms, but disease progression remains uncertain. TV surgery is effective for severe symptoms, but isolated TVR is rare and has a poor prognosis.

## Background

Isolated rheumatic tricuspid valve regurgitation (IRTR) is a rare disorder where blood flows backward from the right ventricle to the right atrium due to a malfunctioning tricuspid valve. It is mainly associated with rheumatic fever, leading to fibrotic alterations and severe regurgitation. IRTR affects only 0.08% of RHD patients [1]. Tricuspid regurgitation is usually asymptomatic and easily tolerated, but isolated cases can cause RHF symptoms like fatigue, congestive hepatopathy, edema, and neck fullness. When tricuspid regurgitation is severe, surgical replacement may be an option [2].

The echocardiography findings of IRTR are not well established due to their rarity related to rheumatic fever. The case presents a rare manifestation of latent RHD, despite not being historically recorded, and echocardiographic screening is the gold standard for diagnosing this rare heart condition [3]. The IRTR, despite its limited evidence, may be more prevalent than initially thought, particularly in regions with a high incidence of RHD. This case is novelty to report IRTR with transthoracal echocardiography (TTE) and transesophageal echocardiography (TEE) for diagnosis confirmation and aims to inform future research on developing new echocardiography criteria for rheumatic heart disease, including tricuspid valves, in addition to mitral and aortic valves.

## Case presentation

A 32-year-old female patient arrived at the cardiology clinic with a primary concern of experiencing difficulty breathing during light physical activity (classified as NYHA functional class III), decreased ability to engage in exercise, history of bilateral leg swelling, accumulation of fluid in the abdomen (ascites), and persistent tiredness throughout 5 months before admission. The woman was a robust homemaker with no medical or surgical conditions in her past. She was the fourth child among four siblings and grew up in overcrowded households with limited access to proper medical care. Her family had a low-income background. Throughout her childhood and teenage years, she often experienced coughs and colds. However, her condition was not given much attention and was not considered important. Medical help was only sought when she experienced symptoms that restricted her daily activities, at age 31 years old, she just started suffering from an episode of chest pain that radiated to her back and left arm and was accompanied by cold sweats. Following a period of one and a half months, she once again encountered chest pain accompanied by perspiration, resulting in her hospitalization in the critical care unit for 8 days.

During the physical examination, her vital signs were within the usual range. The musculoskeletal examination was found to be in good condition, while the respiratory and abdominal investigations yielded no abnormalities. The central venous pressure was judged to be 4 cm above the sternum, and there was slight swelling in the lower limbs. The pericardial examination showed a displaced apex beat at the 6th Inter Costae Space (ICS), 2 cm to the left of the midclavicular line, accompanied by heaves or thrills. The cardiac auscultation revealed normal first and second heart sounds, accompanied by a loud pansystolic murmur IV/VI that was audible across the cardiac regions, particularly at the lower sternal boundary. The murmur intensified during inspiration and there was no presence of pericardial rub.

**Fig. 1:**
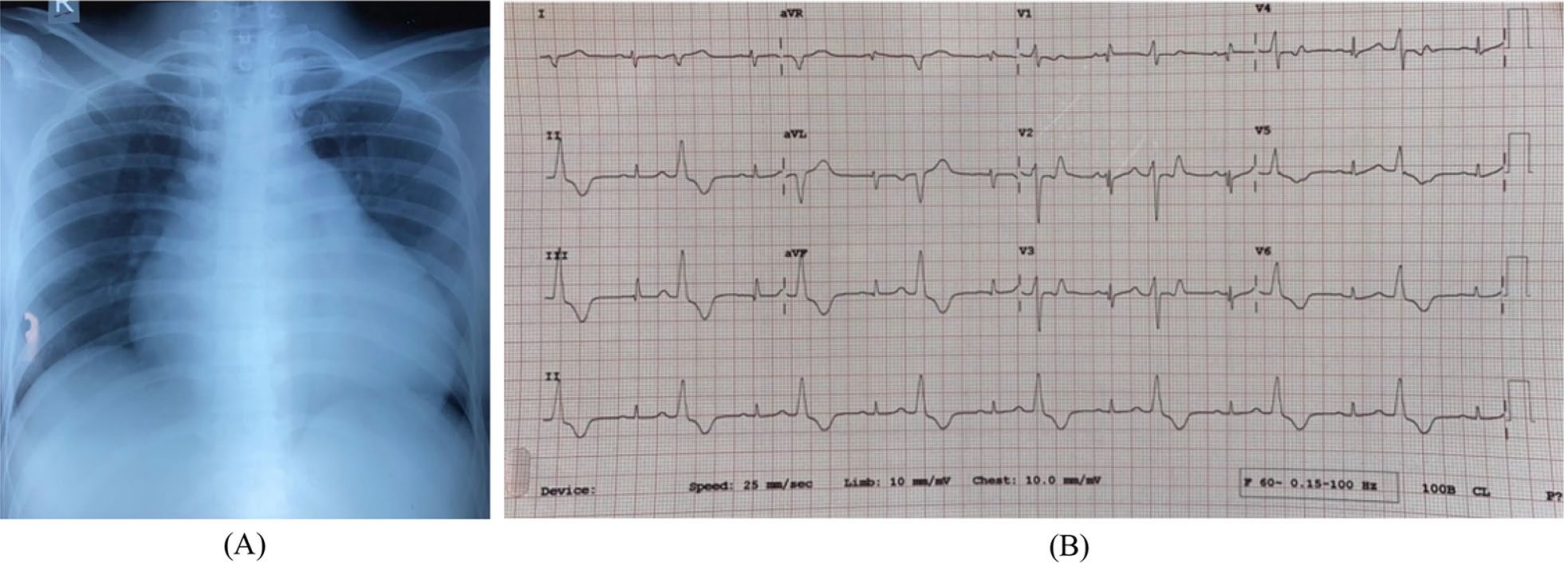
ECG and chest X-ray at initial presentation

Cardiomegaly was observed on the chest radiograph taken on September 11, 2023. The 12-lead electrocardiogram (ECG) conducted on November 11, 2023, indicated a sinus rhythm with a heart rate of 50 beats per minute. The rhythm was regular and demonstrated right axis deviation (RAD) with a counterclockwise rotation. The rotation of the object is occurring around the horizontal axis. The PR interval is measured to be 160 ms and normal QRS duration. Frequent premature ventricular contractions occur in a pattern known as bigeminy, originating from the right ventricular outflow tract (Fig. 1).

**Fig. 2:**
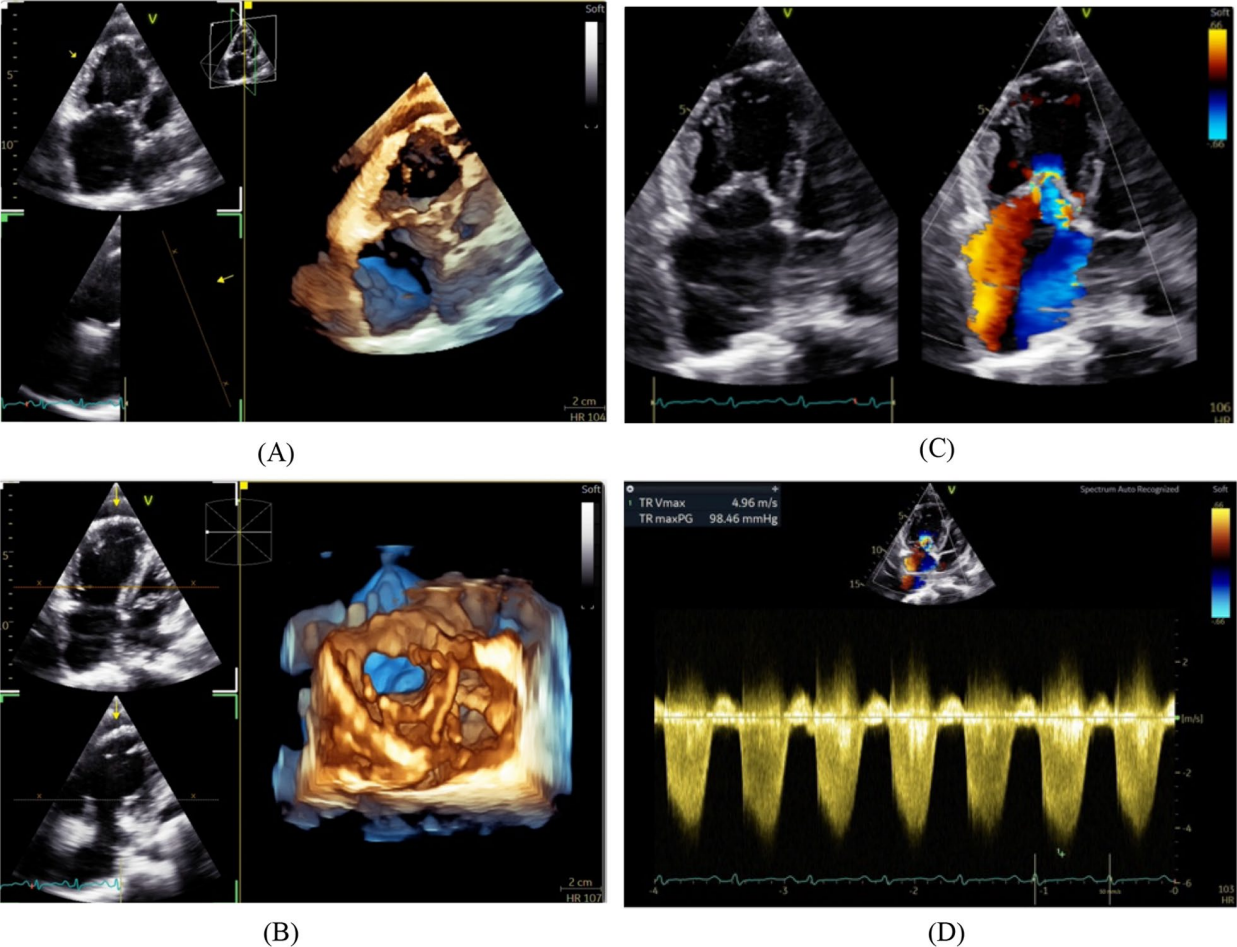
**A**, **B** 3D-TEE: Significant thickening and calcification of all tricuspid valve leaflets, with a partial prolapse suspected in the posterior leaflet; **C** 2D-TTE: The tricuspid valve (TV) is experiencing a significant backflow of blood, as observed using color Doppler imaging. Wall-impinging jet accounts for more than 50% of the right atrium's volume. The vena contracta width measured 0.7 cm, and the TR was predominant in the right atrium, right ventricle, and right atrium exhibited enlargement. **D** 2D-TTE: Continuous wave The Doppler analysis of the TV revealed a jet with a pansystolic pattern, characterized by a dense, semi-triangular form that reached its maximum intensity early. The peak velocity of the jet was measured at 4.9 m/s

**Fig. 3:**
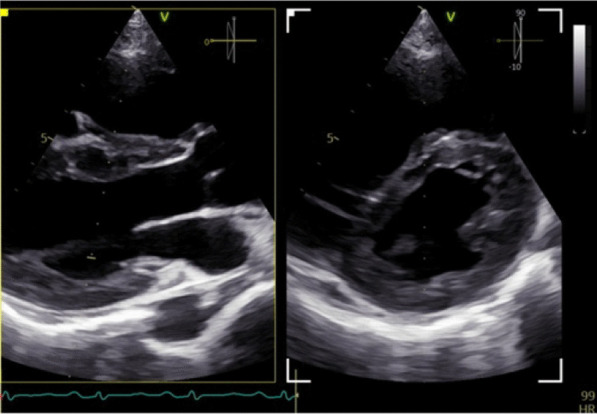
The mitral valve (MV) exhibits normal opening without any indication of leaflet thickness or stenosis. The aortic valve (AV) exhibits a normal anatomical arrangement

From these clinical symptoms and physical examination, cardiologists have suspected congenital heart disease or primary pulmonary hypertension as a differential diagnosis [4, 5]. TTE and 3D-TEE are performed to confirm the diagnosis [6]. The echocardiogram revealed significant thickening and calcification of all tricuspid valve leaflets, with a partial prolapse suspected in the posterior leaflet (Fig. 2). Severe tricuspid regurgitation was observed using the color Doppler, visible in both the 4-chamber and parasternal short axis views. The vena contracta width measured 0.7 cm, and the TR was predominant in the right atrium. An examination of the continuous wave across the TR showed a concentrated, semi-triangular stream of fluid that reached its highest point at the beginning of the contraction phase of the heart, with a speed of 4.9 m per second. The right ventricle and right atrium exhibited enlargement. The PV, MV, and AV exhibited no significant anatomical and functional abnormality, whereas the LV displayed appropriate dimensions and functionality (Fig. 3). There were no findings of pericardial effusion, intra-atrial septal defects, or intra-ventricular septal abnormalities.

The patient received a diagnosis of IRTR due to latent RHD based on heart failure as the worsening complication of RHD and an echocardiographic finding with an undocumented history of ARF [7].

The patient is receiving a daily dose of ramipril 2.5 mg, bisoprolol 2.5 mg, spironolactone 25 mg, and furosemide 40 mg as part of her treatment. The treatment resulted in the alleviation of chest discomfort, dyspnea, and ascites. The patient had a notable improvement in her dyspnea and ascites with the administration of regular medication. However, she still experiences difficulty breathing during light physical activity (classified as NYHA functional class III) therefore she will be included in the surgical conference for consideration of surgical tricuspid valve replacement (TVR).

## Discussion

RHD is the long-term effect of ARF, which is brought on by an autoimmune response to an infection with Group A beta-hemolytic Streptococcal (GAS). Poor hygiene, a crowded home, and limited access to quality medical care are among the predisposing factors that are frequently present in middle-class and lower-class populations [7]. This report illustrates the case of latent RHD due to echocardiographic proof of RHD but no documented history of ARF. Until it manifests in adulthood, latent RHD is typically clinically quiet. More severe RHD consequences, such as heart failure are commonly seen in adults which made the patient admitted to the hospital [7].

Tricuspid regurgitation may arise from an inherent flaw in the valve itself or, more frequently, as a result of an associated issue with the MV. Tricuspid regurgitation rarely occurs alone and is often not detected or causing any symptoms. Patients typically seek medical attention with the onset of heart failure symptoms. Despite normal left-sided valve function, pulmonary pressures, and LV systolic performance, patients with isolated severe TR will have RHF. Increased central venous pressure, visible jugular vein distension, and may occur in organ dysfunction as well as peripheral edema or ascites following the presentation of right heart failure due to backward failure [8]. Right ventricular failure causes systemic congestion due to blood flow issues. Severe cases cause the right side to expand, inhibiting the LV function, resulting in forward failure with low blood pressure and inadequate flow [4, 9]. Exertional dyspnea is caused by low effective operating compliance of the LV due to heightened ventricular interaction with dilated RV. Low perfusion pressure and venous congestion lead to hepatic and renal failure, which demonstrates elevated SGOT, SGPT, urea, and creatinine levels. In cases of chronic RV failure, ECG typically displays RAD due to the enlargement of the RV [8].

Rheumatic carditis is a condition that is marked by inflammation of the endocardium, specifically the valvular endocardium. The predominant manifestation of carditis is the inflammatory condition of the endocardium, which is characterized by valvulitis. Valvulitis primarily affects the mitral valve, resulting in MR [10]. The occurrence of isolated right-sided cardiac valve involvement, particularly TR, without any accompanying MV disease that results in RV failure, is an exceptionally rare discovery, especially during screening. As a result, it is excluded from the guidelines [6].

Due to the high sensitivity of echocardiography, carditis can be ruled out in a patient with a murmur if there is no regurgitation on color Doppler. Furthermore, echocardiography can easily identify murmurs brought on by endocarditis, congenital anomalies, or myxomatous mitral valve disease. The Jones criteria and echocardiogram were concordant (both positive or both negative) 83% of the time in a study of 333 patients with suspected clinical carditis. Patients with “subclinical” or “latent” carditis, in whom an echocardiogram shows characteristics suggestive of valvulitis but the clinical examination is negative, are the subject of controversy. In the Utah outbreak, only 14 out of 74 individuals (19%) had carditis found by echocardiography. Seventeen percent of patients in a meta-analysis with 1700 cases had subclinical carditis as their occurrence. The primary cause of the notable variations in study results is the use of different echocardiographic standards to diagnose carditis. The WHO criteria are as follows: (1) regurgitant jet length > 1 cm; (2) regurgitant jet visualized in at least two planes; (3) mosaic jet with peak velocity > 2.5 m/s; and (4) pansystolic mitral or pandiastolic aortic regurgitation. However, some have suggested raising the minimum jet length of mitral or aortic regurgitation to 2 cm to increase specificity; and still others require additional morphological valvular abnormality to decrease the number of carditis diagnoses [11].

Ten percent of adult instances of TR are related to primary valvular disease. Myxomatous prolapse, atrioventricular abnormalities, and Ebstein’s abnormality are examples of primary TV illnesses that can occur in patients with congenital disease. Trauma-induced flail leaflets, carcinoid, rheumatoid arthritis, and endocarditis are examples of acquired primary diseases [8, 12]. Echocardiography revealed no congenital heart anomalies or primary pulmonary hypertension as the cause. Instead, we discovered IRTR with thickened leaflets, calcification of all leaflets, and partial prolapse posterior leaflets in the nonsignificant abnormalities in the other valves [13].

Rare RHD symptoms such as isolated severe TR are little documented in the literature. The current guidelines regarding echocardiographic characteristics of rheumatic carditis exclusively emphasize aortic and mitral valve abnormalities, disregarding rheumatic TV conditions [6, 10]. We establish that the echocardiographic observations of IRTR exhibit similarities to those documented for rheumatic MR. Through the use of continuous wave Doppler, this case study illustrates how the echocardiographic findings of isolated rheumatic MR and rheumatic TR are similar in terms of leaflet thickening, presence in at least two views, and a pansystolic jet. The high-velocity jet is the outlier, most likely because of the right side of the heart’s low pressure [1].

The majority of individuals with symptomatic TR are treated with diuretics to reduce volume overload and medicines aimed at the underlying illness process. Diuretics can temporarily slow down symptoms, but it is not known if this will change how the disease progresses, especially in those with primary valve disease [2, 8]. TV surgery is therefore the only effective treatment for severe symptoms. European Society of Cardiology Recommendations 2012 Class I recommendation for tricuspid valve surgery for severe primary or secondary TR at the time of left-sided valve surgery (level of evidence C) and symptomatic isolated severe primary TR without evidence of right ventricular dysfunction (level of evidence C) [5]. AHA/ACC Recommendations 2014 Class I recommendation for severe primary or secondary TR at the time of left-sided valve surgery (level of evidence C) and Class II recommendation for severe primary TR in patients unresponsive to medical therapy surgery may be appropriate (level of evidence C) [14]. Whereas 2020 ACC/AHA Guideline for the Management of Patients With Valvular Heart Disease give Class IIa recommendation for isolated tricuspid valve surgery that may be helpful to patients with severe primary TR (Stage D) and right-sided HF signs and symptoms to lessen symptoms and hospital readmissions [15].

The majority of recommendations are intended for patients having simultaneous mitral or aortic surgery. Based on the observation that the TV's size and design do not consistently revert to baseline following the reduction of RV overload, these recommendations have been made. Patients who have severe annular dilatation, a history of unsuccessful TV repair, or abnormalities in the leaflets may need to have their TVR [8]. In this instance, surgical intervention should be taken into consideration as our patient exhibits severe isolated TR along with indications of right heart failure. Transcatheter treatments may manage functional TR but not organic TR like leaflet flail, prolapse, rheumatic disease, carcinoid disease, or endocarditis. Surgical replacement may be preferable due to suspected partial prolapse in the posterior leaflet [8, 16].

Isolated tricuspid valve replacement is a rare procedure with poor outcomes in patients with RHD and previous mitral valve replacements. It is associated with high post-operation problems and poor short and long-term prognosis. Only in specific circumstances after a thorough clinical and hemodynamic examination should it be performed. The scarcity of cases has limited data on its results [17]. Secondary antibiotic prophylaxis has been demonstrated effective in slowing the development of latent rheumatic heart disease [3].

## Conclusions

IRTR is a rare medical condition with limited coverage in current guidelines. It shares echocardiographic characteristics of rheumatic TR that closely resemble those of rheumatic MR, except for the presence of a high-velocity jet. TVR is the only effective treatment for severe symptoms, but isolated TVR is linked to a negative prognosis. This case report aims to inform future research on developing additional echocardiography criteria for RHD by including TV alongside MV and AV.

## Data Availability

Supporting data regarding this publication (patient data), aside from what has already been provided in the paper, can be obtained through the author.

## Abbreviations

AV: Aortic valve
ECG: Electrocardiogram
ICS: Inter costae space
IRTR: Isolated rheumatic tricuspid valve regurgitation
LV: Left ventricle
MV: Mitral valve
MR: Mitral regurgitation
NYHA: New York Heart Association
PV: Pulmonary valve
PVC: Premature ventricular contractions
RAD: Right axis deviation
RHD: Rheumatic heart disease
RHF: Right heart failure
RV: Right ventricle
RVOT: Right ventricular outflow tract
SGOT: Serum glutamic oxaloacetic transaminase
SGPT: Serum glutamic pyruvate transaminase
TEE: Transesophageal echocardiography
TR: Tricuspid regurgitation
TTE: Transthoracal echocardiography
TV: Tricuspid valve
TVR: Tricuspid valve replacement
